# The complete chloroplast genome sequence of the Japanese Camellia (*Camellia japonica* L.)

**DOI:** 10.1080/23802359.2017.1372719

**Published:** 2017-08-30

**Authors:** Seon-Hee Kim, Chung Hyun Cho, Misuk Yang, Seung-Chul Kim

**Affiliations:** a Department of Biological Sciences, Sungkyunkwan University, Suwon, Gyeonggi-do, Republic of Korea;; b AmorePacific Corporation, R&D Center, Yongin-si, Gyeonggi-do, Republic of Korea

**Keywords:** *Camellia japonica*, chloroplast genome, phylogenetic relationships

## Abstract

The complete chloroplast genome sequence of valuable ornamental tree, *Camellia japonica* L. (Theaceae), was determined. The genome size was 156,971 bp in length, containing a pair of 25,798 bp inverted repeat (IR) regions, which were separated by small and large single copy regions (SSC and LSC) of 18,394 and 86,673 bp, respectively. The cp genome contained 134 genes, including 91 coding genes, six rRNA genes, and 37 tRNA genes. The overall GC content of the chloroplast genome was 37.3%. Phylogenetic analysis revealed the position of *C. japonica* being sister to the clade containing *C. crapnelliana* and *C. oleifera* (subgenus *Camellia*).

The genus *Camellia* L., containing more than 200 species, is the largest and economically most important genus in the family Theaceae (Vijayan et al. 2009). It is mainly distributed in East and Southeast Asia and is widely cultivated around the world (Vijayan et al. 2009). Species of the *Camellia* provide many valuable products, including nonalcoholic tea, edible oils, and ornamental plants (Eden 1958; Kondo 1975; Ming 2000; Gao et al. 2005; Zhang et al. 2007; Vijayan et al. 2009). Especially, *C. japonica* is the most important and attractive ornamental plant. In addition, it is one of the important members on forest environment of coastal Japan and the southern and western of Korea Peninsula (Numata 1974; Chung and Kang 1996).

We sampled *C. japonica* in Wimi-ri, Jeju Island, Korea (voucher specimen: SKK1508014001) and obtained the complete chloroplast genome sequences. Sequencing was done using the Illumina HiSeq 2000 (Illumina, San Diego, CA) at Macrogen Inc. (Seoul, South Korea) and assembled by SOAP *de novo* 2 (Luo et al. 2012) and CLC Genomics Workbench v.5.5.1 (CLC Bio, Aarhus, Denmark) with Geneious v.8.1.6 (Biomatters Ltd., Auckland, New Zealand). Gene annotation was done using Blast X, Geneious v.8.1.6 (Biomatters Ltd., Auckland, New Zealand), and then manually corrected for start and stop codons and for intron/exon boundaries. Large and small subunits of ribosomal RNA (rRNA) were identified using RNAmmer 1.2 Server (Lagesen et al. 2007), whereas the transfer RNAs (tRNA) were predicted using ARAGORN v1.2.36 (Laslett and Canback 2004). The complete chloroplast genome of *C. japonica* (GenBank ID: KU951523) was then aligned with 14 representative congeneric species of *Camellia* using MAFFT v.7 (Katoh and Standley 2013). Maximum likelihood (ML) analysis was conducted based on the concatenated 128 chloroplast coding genes using IQ-TREE v.1.4.2 (Nguyen et al. 2015).

The complete chloroplast genome of *C. japonica* has a total length of 156,971 bp, which is composed of large single copy (LSC) region of 86,673 bp, small single copy (SSC) region of 18,394 bp and two inverted repeat (IRa and IRb) regions of 25,952 bp each. The GC contents of the entire chloroplast genome were 37.3%, and those of LSC, SSC, and IR regions were 35.3%, 34.6%, and 43%, respectively. The chloroplast genome included 134 genes, including 91 protein-coding genes, six rRNA genes, and 37 tRNA genes. A gene loss or genome structural change was not detected. The *ycf*1 gene in the junction region of IRb and SSC was the only pseudogene found, which was created by incomplete duplication of the normal copy of *ycf*1 in the IRa and SSC junction region.

The phylogenetic relationship of *C. japonica* was inferred by its comparison with other 14 chloroplast genomes in *Camellia*, representing two subgenera and six sections (Ming 2000). The ML tree showed that *C. japonica* (sect. *Camellia*) was sister to the clade containing *C. crapnelliana* (sect. *Heterogenea*) and *C. oleifera* (sect. *Oleifera*) of subgenus *Camellia* (Figure 1). Most sections and two subgenera were not monophyletic, reflecting highly specious and reticulating lineage of genus *Camellia* (Vijayan et al. 2009).

**Figure 1.**
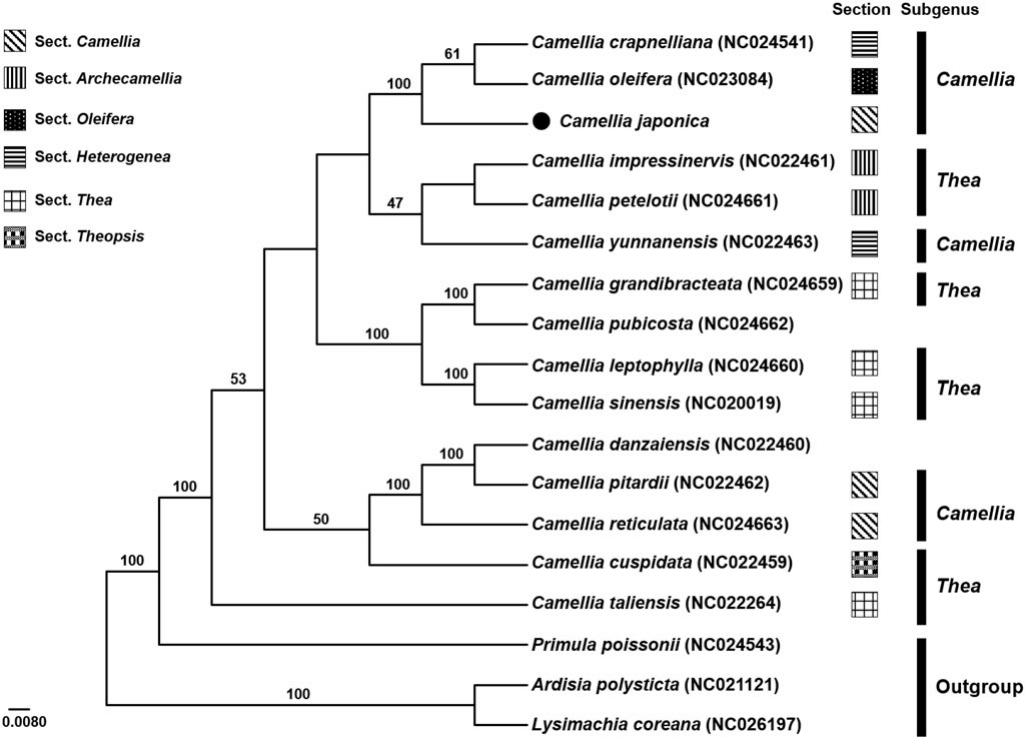
Maximum likelihood tree of the genus *Camellia* based on representative complete chloroplast genome sequences, including *C. japonica* (GenBank ID: KU951523) sequenced in this study. The bootstrap support values are shown above the branches. Three representative taxa of Ericales (*Primula poissonii*: NC024543, *Ardisia polysticta*: NC021121, *Lysimachia coreana*: NC026197) were used as outgroups.
